# Endoscopic vacuum therapy in the upper gastrointestinal tract: when and how to use it

**DOI:** 10.1007/s00423-022-02436-5

**Published:** 2022-01-18

**Authors:** Christian A. Gutschow, Christoph Schlag, Diana Vetter

**Affiliations:** 1 Department of Visceral and Transplant Surgery, University Hospital Zurich, Rämistrasse 100, 8091 Zurich, Switzerland; 2 Department of Gastroenterology and Hepatology, University Hospital Zurich, Rämistrasse 100, 8091 Zurich, Switzerland

**Keywords:** Endoscopic vacuum therapy, Negative pressure therapy, Anastomotic leakage, Esophageal perforation, Esophagectomy; Gastrectomy

## Abstract

**Background:**

Endoscopic vacuum therapy (EVT) has emerged as a novel treatment option for upper gastrointestinal wall defects. The basic principle of action of EVT entails evacuation of secretions, removal of wound debris, and containment of the defect. Furthermore, there is increasing evidence that EVT reduces interstitial edema, increases oxygen saturation, and promotes tissue granulation and microcirculation. Various devices, such as macroporous polyurethane sponge systems or open-pore film drains, have been developed for specific indications. Depending on the individual situation, EVT devices can be placed in- or outside the intestinal lumen, as a stand-alone procedure, or in combination with surgical, radiological, and other endoscopic interventions.

**Purpose:**

The aim of this narrative review is to describe the current spectrum of EVT in the upper gastrointestinal tract and to assess and summarize the related scientific literature.

**Conclusions:**

There is growing evidence that the efficacy of EVT for upper GI leakages exceeds that of other interventional treatment modalities such as self-expanding metal stents, clips, or simple drainages. Owing to the promising results and the excellent risk profile, EVT has become the therapy of choice for perforations and anastomotic leakages of the upper gastrointestinal tract in many centers of expertise. In addition, recent clinical research suggests that preemptive use of EVT after high-risk upper gastrointestinal resections may play an important role in reducing postoperative morbidity.

## Introduction and definition

Endoluminal vacuum therapy (EVT) evolved from external use of this technique, which was implemented by plastic surgeons some 15 years ago for the treatment of infected and ischemic wounds [1]. It is noteworthy that EVT was initiated by surgical endoscopists, who—from their general surgical background—were aware of the great potential of external vacuum therapy. Initial steps with EVT were made in the lower gastrointestinal (GI) tract as a treatment for anastomotic leakage (AL) after rectal surgery [2], and the first application in the foregut was reported in 2007 in a case with AL after gastrectomy [3].

Vacuum therapy can be defined as a technique in which the wound compartment is subjected to a pressure lower than that of the atmosphere, which requires a suction pump and an airtight seal. In EVT, this seal is self-establishing because the negative pressure causes collapse of the surrounding tissue, isolating the wound area from the atmosphere and creating a closed negative pressure environment.

Others prefer the term “negative pressure therapy” (NPT) instead of “vacuum therapy,” arguing that negative pressure does not exactly meet the definition of a vacuum [4]. However, a perfect vacuum is a theoretical construct that cannot be achieved even under ideal laboratory conditions and can be considered a philosophical ontological concept like “The Nothingness” [5, 6]. Moreover, “NPT” is often used as a generic term for all therapies that use suction, including simple drainage with single- or double-lumen drains and tubes. Therefore, we believe that the term “EVT” is very appropriate, as it corresponds well to both the common technical definition of vacuum (“the state of a gas when the pressure of the gas is lower than the lowest atmospheric pressure prevailing on the surface of the earth”) [7] and the physical conditions that prevail during therapy.

Until recently, no certified technical solution for EVT in the foregut was available on the market, and devices had to be self-designed and -manufactured. In this regard, the introduction of the EsoSponge® system (B.Braun Melsungen AG, Melsungen, Germany) in 2014 was an important step towards standardization.

Due to the promising clinical results and growing scientific evidence [8–11], EVT is becoming increasingly recognized as an important clinical tool and has evolved in many—especially European—centers of expertise as the standard treatment for foregut wall defects. In this context, this review aims at summarizing the current spectrum of EVT in the foregut and at providing a comprehensive overview of the actual literature.

## Indications for EVT in the upper GI tract

Indications for upper GI EVT entail the whole spectrum of transmural wall defects of the esophagus and the esophago-gastric junction, with the published evidence focusing on the treatment of suture line leakage after oncological upper-GI resections [12] and bariatric procedures [13], iatrogenic perforations, but also on spontaneous ruptures (Boerhaave’s syndrome) [14]. Depending on the individual situation, EVT is performed as a stand-alone procedure or in combination with surgical, endoscopic, or radiological interventions. In this context, several retrospective studies have demonstrated high success rates of EVT with low complication rates [15–19]. In addition, compared with endoscopic stenting, EVT was associated with higher AL closure rates, shorter treatment duration, and lower mortality in several meta-analyses of retrospective studies [11, 14, 20, 21]. Other potential indications for EVT, which require specific endoscopic techniques and drain types, include duodenal wall defects [22–27] and pancreatic fistula [4, 28–30].

In contrast, the applicability of EVT is limited in the proximal esophagus and hypopharynx, as the proximal location prevents airtight separation from the atmosphere with creation of a contained negative pressure environment. Similarly, gastric lesions, especially those in the fundus and corpus, are mostly unsuitable for EVT treatment owing to difficulties in establishing a sealed negative pressure compartment in large hollow organs. Furthermore, the need of repetitive endoscopic procedures for EVT exchange (usually every 3–5 days) and keeping patients “nil by mouth” for the duration of EVT is not tolerated by every patient and has to be considered as potential drawback.

## Mechanism of action

The basic principle of EVT is to create a negatively pressurized compartment that promotes shrinkage, containment, cleaning, and granulation of the infected wound area. Depending on the intensity of the applied negative pressure and the mechanical properties of the device and the surrounding wound tissue, the vacuum causes collapse of the wound cavity, a phenomenon for which the term “macro-deformation” has been coined. In this context, a negative pressure of 125 mmHg has been shown to reduce the volume of a polyurethane sponge by up to 80% [1, 31–33]. On the other hand, no significant difference in wound diameter has been evidenced in a porcine model after increasing the negative pressure from − 75 mmHg to − 175 mmHg [34], and it may thus be concluded that a relatively low negative pressure is generally sufficient for adequate macro-deformation.

**Fig. 1:**
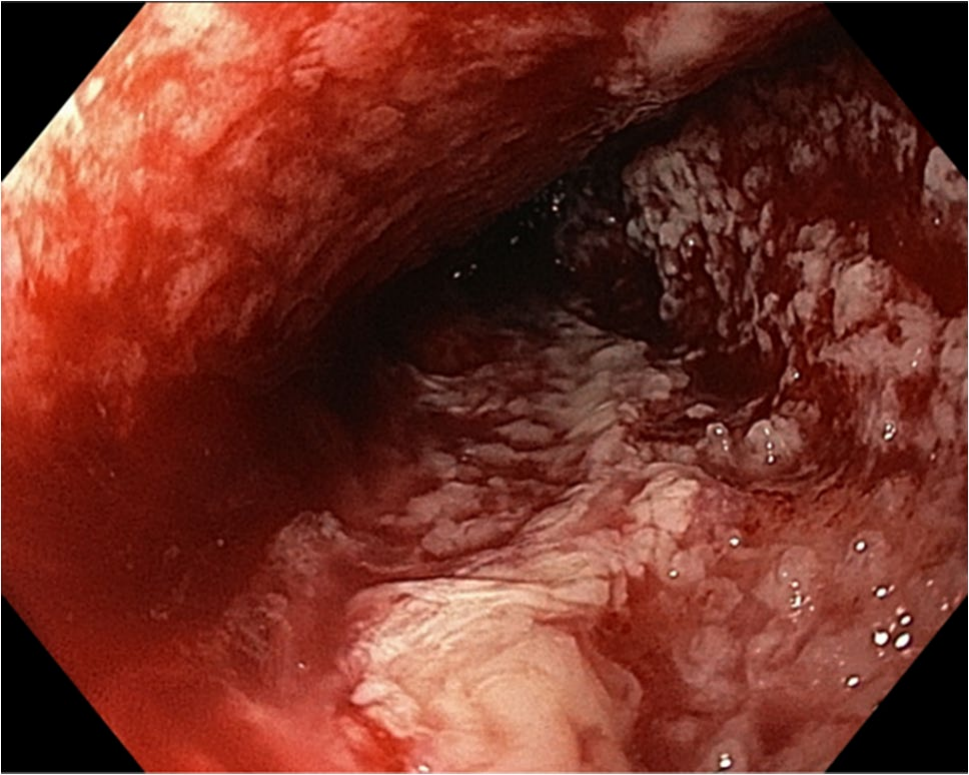
Small nodules of granulation tissue (micro-deformation) are visible after removal of an EVT device. The extent of micro-deformation depends on various factors such as the porosity of the connecting material, the intensity of the negative pressure, and the properties of the wound surface

The direct effect of negative pressure to the wound surface has been termed “micro-deformation.” Micro-deformation can be observed as small granulation tissue nodules after removal of the porous connecting material (Fig. 1). The pathophysiology leading to micro-deformation is not completely understood and involves complex mechanisms of wound healing and cell proliferation [35].

Other mechanisms of action of EVT include drainage of infected fluids, removal of debris and microorganisms, and reduction of interstitial edema. In addition, there is increasing evidence that EVT promotes microcirculation and oxygen saturation via angiogenesis caused by modulated vascular endothelial growth factor expression [36, 37].

Similar to interventional stenting or clip placement, EVT is able to close intestinal wall defects and to prevent further contamination. However, a significant advantage compared with stenting is that the infected cavity behind the dehiscence is drained with the same procedure, eliminating the need for additional external drainage in many cases. Moreover, intestinal wall defects that communicate with other body cavities and spaces can be effectively converted into contained situations, which is of paramount importance in sepsis treatment caused by leakage.

## Devices used for EVT

### Basic principles and considerations

As for external vacuum therapy, the negative pressure is transferred to the wound area via specific connecting materials such as porous sponges or customized films. The material is attached to the distal end of a pressure-resistant plastic tube, which is routed externally and connected to a pump generating negative pressure. Unlike for applications in external wounds, which require the treated area to be sealed with an airtight film, this is not necessary with EVT, as the surrounding tissue collapses around the connecting material and automatically isolates the area of interest from the atmosphere, creating a contained negative pressure environment.

### Polyurethane foam-based drains

EVT is usually performed with polyurethane sponges [1]. One must bear in mind that commercially available PU sponges have variable mechanical properties in terms of porosity and density. Generally, macroporous, low-density sponges are preferred because of the greater debriding capacity and the stronger contraction under negative pressure, which leads to a more pronounced shrinkage (macro-deformation) of the wound cavity. On the other hand, low-density macroporous sponges are more difficult to put in place due to their size. They also grow into the granulation tissue more easily and are therefore more difficult to remove.

**Fig. 2:**
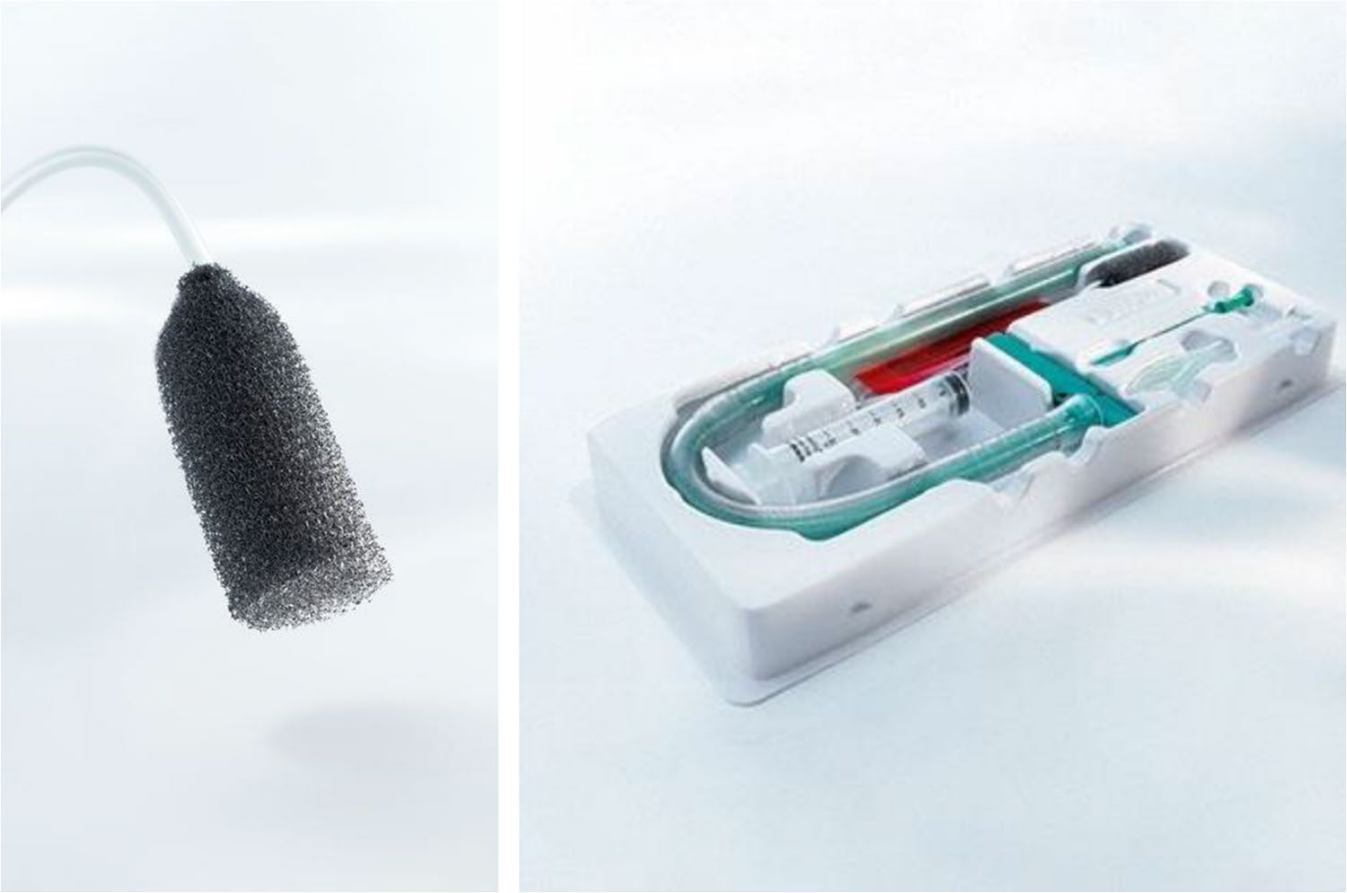
The EsoSPONGE® device (B.Braun) consists of a low-density macroporous PU foam fitted to a pressure-resistant plastic tube. It is supplied with a specifically designed insertion set using an overtube

PU sponge drains for EVT can be self-manufactured by combining readily available PU foams from vacuum therapy for external wounds with conventional naso-gastric tubes. Since 2014, the EsoSPONGE® (B. Braun Melsungen AG, Melsungen, Germany) is approved as a medical device and commercially available in Europe. The EsoSPONGE® features a low-density macroporous PU foam fitted to a pressure-resistant plastic tube. It is supplied with a specifically designed insertion set using an overtube (Fig. 2). In our experience, this insertion set represents a groundbreaking advantage as it both facilitates and standardizes the procedure.

### Open-pore film drains

**Fig. 3:**
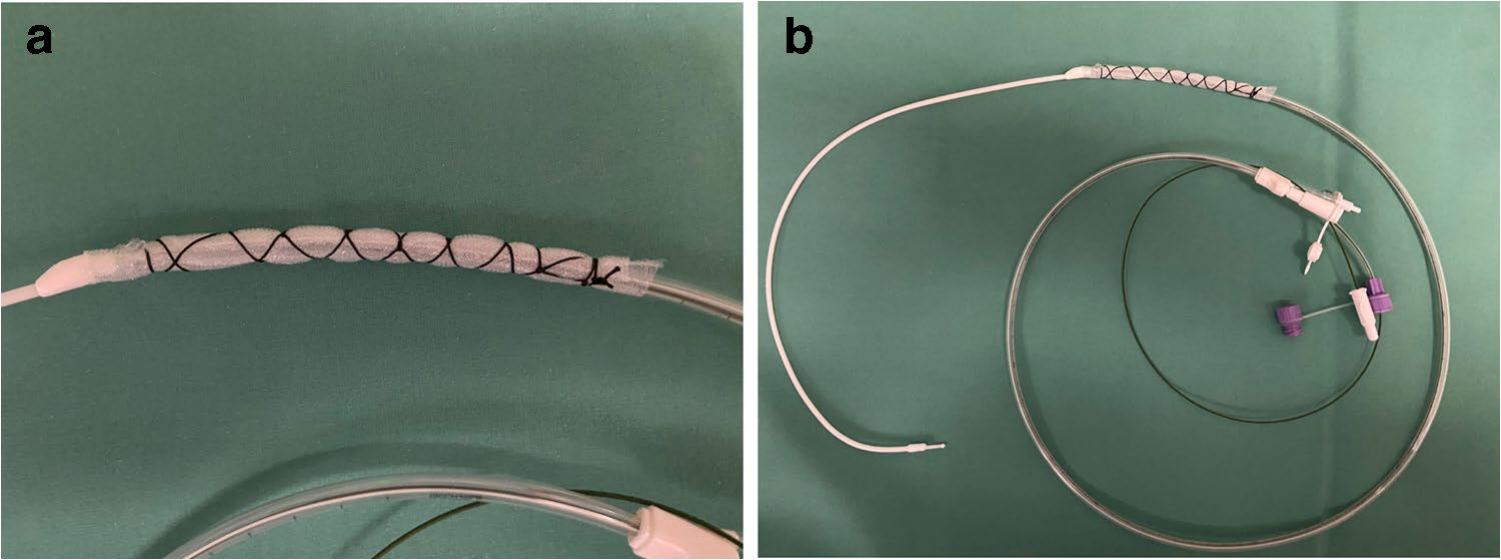
**a** and **b** A self-manufactured open-pore foil drainage for use in the upper GI tract. The perforated part of a three-lumen jejunal feeding and gastric decompression tube (Freka® Trelumina, Fresenius Kabi Deutschland GmbH, Bad Homburg, Germany) is wrapped with a double-layer open-pore film (Suprasorb® CNP Drainage Film, Lohmann & Rauscher International GmbH & Co, Rengsdorf, Germany).

**Fig. 4:**
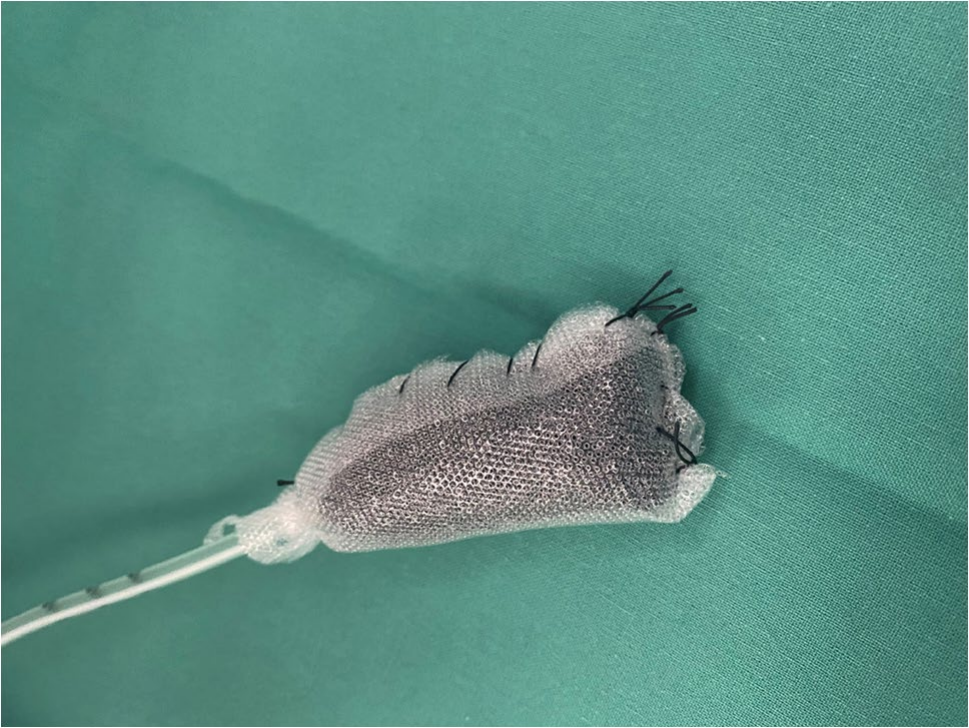
A PU foam drain coated with an open-pored film (Suprasorb® CNP Drainage Film, Lohmann & Rauscher International GmbH & Co, Rengsdorf, Germany) to reduce device ingrowth into vulnerable tissues

**Fig. 5:**
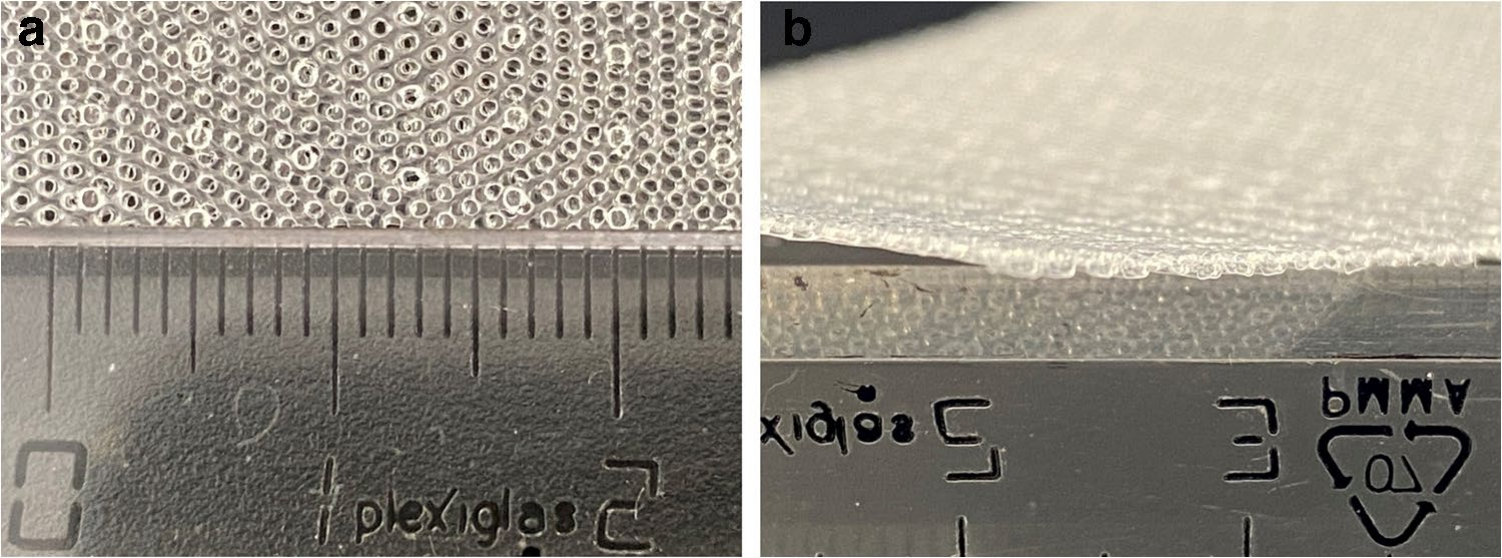
**a** and **b** Double-layered open-pored film (Suprasorb® CNP Drainage Film, Lohmann & Rauscher International GmbH & Co, Rengsdorf, Germany) as used by our team for open-pore foil drains.

For certain indications, permeable films have significant advantages as a connection material compared with PU foam-based drains. So-called “open-pore film drains,” in which the perforated area of the drain is directly wrapped with an open-pored film, are easier to put into place due to their smaller diameter and have the advantage of less adherence to the wound cavity, which also facilitates removal (Fig. 3a,b). As an alternative, the open-pored films can also be used to coat the PU foam in order to reduce tissue ingrowth [28, 38] (Fig. 4). Open-pore film drains for EVT were popularized by G. Loske, who developed a range of different drain types for various indications at the gastrointestinal tract [4]. Based on those publications and suggestions, we use a double-layered open-pored film (Suprasorb® CNP Drainage Film, Lohmann & Rauscher International GmbH & Co, Rengsdorf, Germany), which has been specifically designed and approved for vacuum therapy in open abdominal wounds (Fig. 5a,b) [39, 40]. The Suprasorb® CNP drainage film has a very high permeability and allows for superior fluid transport compared with other commercially available products as shown in a recent randomized trial [41].

### Stent-over-Sponge (SOS) procedure and VacStent®

The stent-over-sponge (SOS) procedure is a technical variant of EVT that combines PU sponges with covered self-expanding metal stents (SEMS). The SOS treatment was introduced at the Zurich University Hospital in 2013 [42], and our experience in patients with upper gastrointestinal leaks was recently published [43]. Compared with EVT using PU sponges only, SOS has several advantages: First, the SEMS keeps the gastrointestinal lumen open after sponge insertion, allowing oral fluid or food intake. Second, the SEMS both seals the sponge and secures its position, which optimizes the direction and the effect of the negative pressure. Third, covered stents isolate the PU foam from saliva and other intestinal secretions, which otherwise may clog its pores with subsequent loss of function. Finally, the negative pressure between the PU foam and the SEMS might also reduce the risk of stent migration. In our experience, the SOS technique is particularly helpful in patients with relevant extraluminal cavities. In this situation, the sponge can be placed directly in the abscess cavity, which is then sealed with the SEMS. Obviously, a major limitation of the SOS technique is the relatively high cost of the procedure, particularly if multiple device changes are required.

**Fig. 6:**
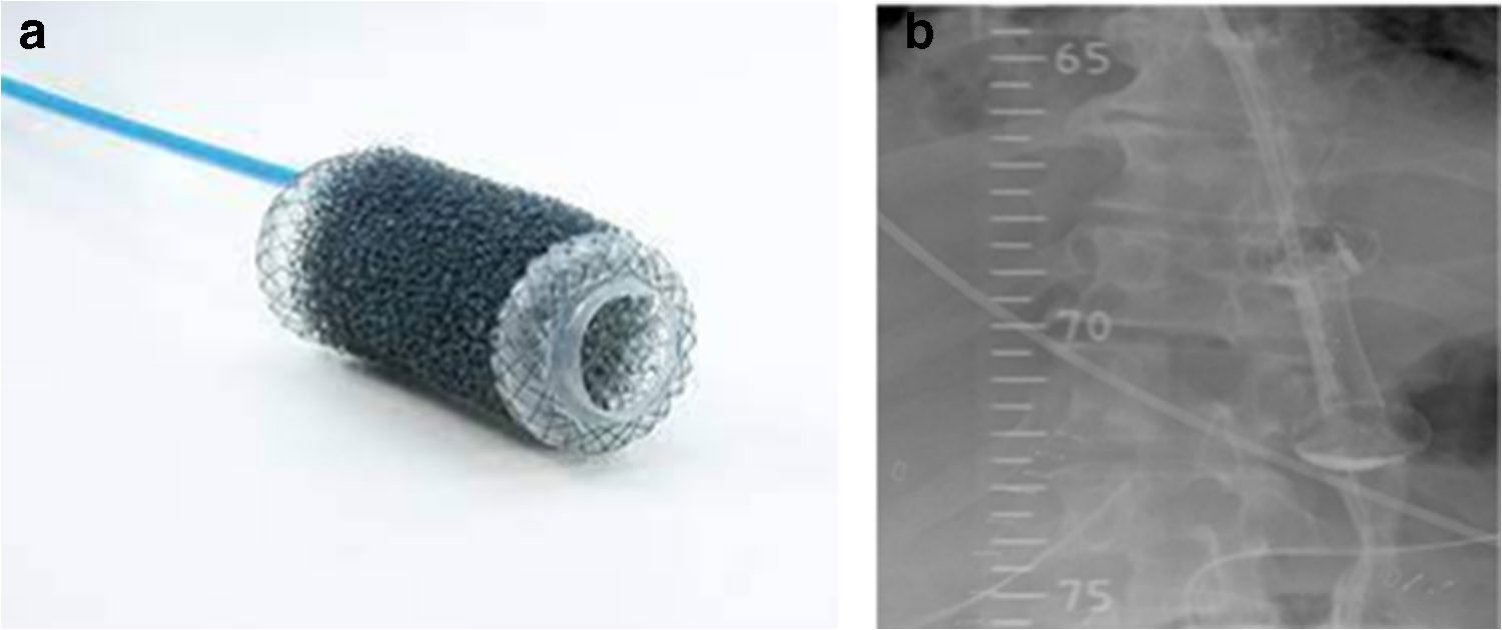
**a** and **b** The VacStent® (VAC Stent Medtec AG, Steinhausen, Switzerland) is specifically designed for EVT and combines a cylindrical PU foam with a covered SEMS. **b** shows a contrast radiography of patient with perforation of the esophago-gastric junction treated with a VacStent®

The VacStent® (VAC Stent Medtec AG, Steinhausen, Switzerland) is a new device for EVT. Like in the SOS procedure, the VacStent® combines PU foam with a covered SEMS, however in a pre-manufactured setup (Fig. 6a,b). The available evidence for this new system is still very limited with two recent case series [44, 45]. Unlike the SOS approach, which allows extra- and intraluminal placement of PU sponges, the VacStent® is only suitable for intraluminal EVT due to the cylindrical shape of the PU foam.

### Electronic pumps and negative system pressure

Preclinical basic research focusing on the underlying mechanisms of EVT is still very limited. In particular, there are no accepted standards for ideal treatment duration and optimal negative system pressure, and these parameters must be selected empirically. Various electronically controlled pumps for vacuum therapy that generate variable negative pressures as low as − 200 mmHg are currently commercially available. However, most devices on the market are specifically designed for external vacuum therapy and are equipped with double-lumen connection systems for pressure monitoring and leakage control that are not compatible with the single-lumen tubing of an EVT device.

In our hands, the electronic Thopaz® vacuum pump from Medela (Medela Healthcare, Baar, Switzerland) has proven a reliable and effective solution. Originally designed for use with chest tubes, the pump produces a maximum negative pressure of − 75 mmHg and tolerates air leaks of up to 2000 ml/min. The system comes with a single-lumen, pressure-resistant tubing that conveniently connects with all types of EVT devices. All relevant information such as negative pressure, leakage, and the amount of liquid collected is continuously monitored and can be accessed via an USB port. In our hands, the relatively low maximum negative pressure of − 75 mmHg has proven sufficient to promote macroscopically visible tissue granulation (micro-deformation), and removal of the sponge without residua is usually unproblematic.

## Management of upper GI wall defects with EVT

When a defect in the upper intestinal wall is suspected, our strategy is to first assess the situation with both contrast-enhanced CT scan and endoscopy. The combination of radiological and endoscopic modalities allows an optimal assessment of potential extraluminal contaminations and a detailed macroscopic luminal evaluation of the wall dehiscence. In our view, endoscopy is clearly indicated even in the early postoperative setting, because of its important diagnostic value combined with effective treatment options. The fear that endoscopy may cause leakage in a fresh and fragile anastomosis is not supported by current scientific evidence [46, 47] and does not outweigh its pivotal advantages regarding early endoscopic assessment and therapy.

Sealing of the intestinal wall defect plus debridement, drainage, and containment of extraluminal contaminations are the paramount therapeutic goals in case of intestinal leakage. It is our policy to always pursue the least invasive therapeutic strategy. In this regard, EVT offers many advantages over conventional approaches, and we found that even severe cases are often suitable for EVT.

**Fig. 7:**
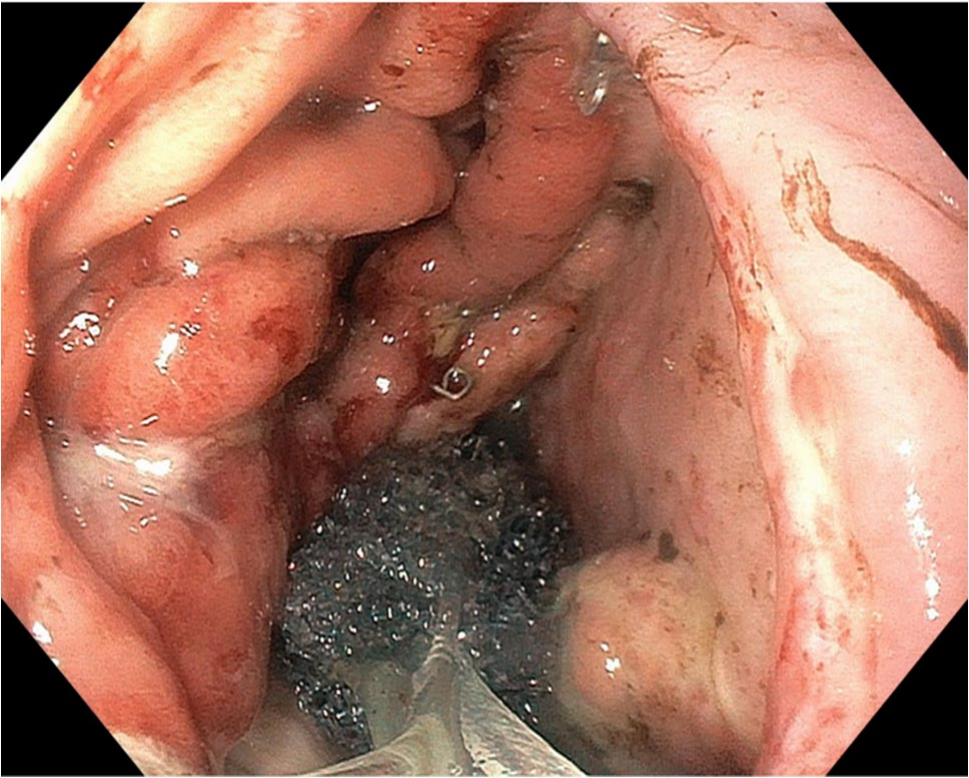
A PU sponge is cut to fit and placed extraluminally/intracavitary with a small extension into the esophageal lumen to drain the abscess cavity and seal the dehiscence, but also to allow free flow of saliva

In general, EVT devices can be placed inside or—in cases with extraluminal abscess or empyema—outside the esophageal lumen. If no relevant extraluminal contamination is present, luminal-only placement of the EVT device is often sufficient. In contrast, relevant extraluminal abscess cavities and/or transmural ischemia require more individualized treatment strategies, often combining endoscopic, radiological, and surgical interventions. For smaller extraluminal cavities, the PU sponge should be trimmed to fit and placed intracavitary with a small extension into the lumen to both drain the cavity and seal the dehiscence, but also to allow free passage of saliva (Fig. 7). If the defect in the foregut wall is too small to permit endoscopic access to an extraluminal abscess cavity, the opening should be widened until adequate extraluminal debridement and device placement are possible. In this context, large extraluminal cavities may require additional over-stenting (SOS procedure) to achieve complete sealing of the wall defect.

It is important to mention that some experts advise against extraluminal placement of EVT devices in the mediastinal compartment, arguing that there is a risk of hemorrhage and bronchopulmonary fistula, especially if the abscess cavity is located close to major vascular structures or airways. Nevertheless, in our own approach, extraluminal EVT is seen less critically because major bleeding or fistula generally occurs very rarely. Furthermore, due to the rather dense mediastinal anatomy, vascular and/or tracheobronchial structures are practically always located in the vicinity of paraesophageal abscess cavities, so the advantages and disadvantages of extraluminal EVT must be carefully weighed in each situation. In order to reduce the risk of hemorrhage and airway fistula during extraluminal EVT treatment, we recommend wrapping the sponge with a non-adhesive, permeable foil (Fig. 4).

## Preemptive EVT to reduce morbidity after esophagectomy

A novel and potentially groundbreaking concept is the application of EVT technology in a preemptive setting (pEVT) with the aim of preventing AL and reducing postoperative morbidity. In a porcine model, intraoperative application of pEVT with PU foam drains in esophago-gastric anastomoses with intentional defects resulted in complete healing [48]. Furthermore, it has been shown that EVT can be an effective salvage strategy to avert AL in early postoperative anastomotic ischemia and imminent leakage [49]. Considering the high incidence and the deleterious effects of AL, our group has recently demonstrated the clinical efficacy and feasibility of pEVT with PU foam drains in patients undergoing minimally invasive esophagectomy with excellent patient safety outcome parameters [47, 50], and we have currently initiated a randomized controlled trial comparing pEVT with standard postoperative care in high-risk patients undergoing minimally invasive Ivor Lewis esophagectomy [51]. Similarly, prevention of reflux and protection of the anastomotic area from duodeno-gastric juices using double-lumen open-pored foil drains is being investigated by other groups [40].

## Summary

EVT offers the option of organ-preserving, minimally invasive treatment even in catastrophic situations that would otherwise require major and potentially mutilating surgical procedures. Consequently, although published evidence is still quite limited, EVT has become the treatment of choice for foregut wall defects in many referral centers.

Continued technical advancement has led to the development of a number of different EVT devices and techniques such as open pore film drains, the SOS procedure, or the VacStent®. These new devices have significantly expanded indications for EVT, including duodenal wall defects and pancreatic fistula. Another new aspect of EVT that deserves our attention is its preventive use to reduce AL rates and overall morbidity after major foregut surgery.

EVT is not a “one-size-fits-all” solution but should rather be seen as a range of complementary tools, which refine the established therapeutic armamentarium. In contrast to traditional endoscopic-interventional approaches such as SEMS or clips, EVT combines well-established surgical concepts such as debridement and drainage with the advantages of endoscopy and is therefore an ideal treatment for foregut wall defects with relevant extraluminal contamination. On the other hand, foregut dehiscence without relevant extraluminal involvement may still be adequately treated by simply sealing the defect with SEMS or clips. In this context, we look forward to the results of the ongoing phase 2 randomized trial (ESOLEAK) that compares SEMS with EVT for the treatment of anastomotic leakage after Ivor Lewis esophagectomy [52].

In conclusion, the prerequisite for successful treatment of potentially septic and fragile patients with foregut leakage is a rigorous clinical, radiological, and endoscopic assessment of the individual situation, combined with detailed knowledge and availability of the full spectrum of current therapeutic concepts. In this context, EVT has evolved to a tool of pivotal importance. As some patients require long-term complex management, close interdisciplinary collaboration between visceral surgeons, interventional endoscopists, radiologists, anesthesiologists, and intensive care medicine is imperative, and we are convinced that such patients are best treated in specialized centers providing optimal care conditions.

## Data Availability

Not applicable**.**
